# Nanostructure Lipid Carrier of Curcumin Co-Delivered with Linalool and Geraniol Monoterpenes as Acetylcholinesterase Inhibitor of *Culex pipiens*

**DOI:** 10.3390/molecules29010271

**Published:** 2024-01-04

**Authors:** Ibrahim Taha Radwan, Nirvina Abdel Raouf Ghazawy, Abeer Mousa Alkhaibari, Hattan S. Gattan, Mohammed H. Alruhaili, Abdelfattah Selim, Mostafa E. Salem, Eman Alaaeldin AbdelFattah, Heba M. Hamama

**Affiliations:** 1 Supplementary General Sciences Department, Faculty of Oral and Dental Medicine, Future University in Egypt, Cairo 11835, Egypt; 2 Department of Entomology, Faculty of Science, Cairo University, Giza 12613, Egypt; 3 Department of Biology, Faculty of Science, University of Tabuk, Tabuk 71491, Saudi Arabia; 4 Department of Medical Laboratory Sciences, Faculty of Applied Medical Sciences, King Abdulaziz University, Jeddah 22254, Saudi Arabia; hsqattan@kau.edu.sa; 5 Special Infectious Agents Unit, King Fahad Medical Research Center, King AbdulAziz University, Jeddah 21362, Saudi Arabia; 6 Department of Clinical Microbiology and Immunology, Faculty of Medicine, King AbdulAziz University, Jeddah 21589, Saudi Arabia; 7 Department of Animal Medicine (Infectious Diseases), Faculty of Veterinary Medicine, Benha University, Toukh 13736, Egypt; 8 Department of Chemistry, College of Science, Imam Mohammad Ibn Saud Islamic University (IMSIU), P.O. Box 90950, Riyadh 11623, Saudi Arabia

**Keywords:** curcumin, *Culex pipeins*, nanostructure lipid carrier, enzyme activity, molecular docking

## Abstract

(1) Background: A molecular hybridization docking approach was employed to develop and detect a new category of naturally activated compounds against *Culex pipiens* as acetylcholinesterase inhibitors via designing a one-pot multicomponent nano-delivery system. (2) Methods: A nanostructure lipid carrier (NLC), as a second generation of solid lipid nanoparticles, was used as a carrier to deliver the active components of curcumin (Cur), geraniol (G), and linalool (L) in one nanoformulation after studying their applicability in replacing the co-crystallized ligand imidacloprid. (3) Results: The prepared nanostructure showed spherical-shaped, polydisperse particles ranging in size from 50 nm to 300 nm, as found using a transmission electron microscope. Additionally, dynamic light scattering confirmed an average size of 169 nm and a highly stable dispersed solution, as indicated by the zeta potential (−38 mV). The prepared NLC-Cur-LG displayed competitive, high-malignancy insecticidal activity against fourth instar *C. pipiens* with an elevated rate of death of 0.649 µg/mL. The treatment, due to the prepared nanostructure, affects oxidative stress enzymes, e.g., hydrogen peroxide (4 ppm), superoxide dismutase (SOD) (0.03 OD/mg), and protein carbonyl (0.08 OD/mg), and there are observable upward and downward fluctuations when using different concentrations of NLC-Cur-LG, suggesting significant problems in its foreseeable insecticidal activity. The acetylcholinesterase activity was assessed by an enzyme inhibition assay, and strengthened inhibition occurred due to the encapsulated NLCs (IC_50_ = 1.95 µg/mL). An investigation of the gene expression by Western blotting, due to treatment with NLC-Cur-LG, revealed a severe reduction of nearly a quarter of what was seen in the untreated group. As a preliminary safety step, the nanoformulation’s toxicity against normal cell lines was tested, and a reassuring result was obtained of IC_50_ = 158.1 µg/mL for the normal lung fibroblast cell line. (4) Conclusions: the synthesized nanoformulation, NLC-Cur-LG, is a useful insecticide in field conditions.

## 1. Introduction

Vector-borne diseases have a significant impact on animal health and cause economic losses in livestock. To control insects, we seek a powerful substitute for synthetic chemical insecticides. In the past, natural compounds derived from plants were employed in animal diets for the beneficial effects of their antimicrobial flavoring agents in addition to some other nutritional impacts [1]. Several studies have discussed the effectiveness of natural-based plant products and or by-products, such as citrus peels in livestock, and other research has inspected active ingredients derived from their main sources using extraction methods, including flavonoids and polyphenols, because of their efficacy in their single-form rather than in the plant matrix [2].

Curcumin is a high-molecular-weight, polyphenolic, hydrophobic phytochemical substance found in the rhizomes of turmeric (curcuma resp.), a Zingiberaceae family member that is widely farmed in Asian nations [3]. Turmeric contains an ingredient known as curcumin that gives it its yellow color [4]. Turmeric is composed of 6–8% proteins, 60–70% carbohydrates, 3–7% minerals, 5–10% lipids, and 6–13% moisture by weight [5]. However, more than 50 compounds of structurally similar chemicals make up 3–5% of the curcuminoids. Curcumin, demethoxycurcumin, and bisdemethoxycurcumin are the three major substances [6]. Due to its putative anti-inflammatory and antioxidant properties [7,8], curcumin has been widely verified to be active in treating different conditions, including anxiety, hyperlipidemia, inflammatory and oxidative disorders, arthritis, metabolic syndrome, and cancers, including lung, bladder, and breast cancers [9,10,11,12,13].

Also, curcumin has been used as a potential antimicrobial agent as a result of its wide activity against many bacteria, fungi, and virus microorganisms [14,15]. Numerous in vivo, in vitro, and in silico research projects have revealed that curcumin influences several signaling pathways, like NF-B, MAPK/ERK, and STAT, which are linked to distinct cancer malignancies [16]. Additionally, curcumin has nutritional and pesticidal qualities that enhance the performance of poultry and cattle animals in terms of production, as well as broad-spectrum action on insects that harm agronomic products and can transmit illnesses to people. Another new scope of curcumin study is the enhancement of animal nutrition through the improvement of meat quality, weight gain, and immune system acceleration to keep poultry and cattle animals disease-free [17]. The potential of curcumin to eradicate insects is a critical topic of investigation. Curcumin has a unique ability to preserve crops, act as a possible pesticide, and supply the agricultural sector with natural insecticides that are safe to use on insects by inducing a range of insect pest growth-inhibiting effects [18,19]. Curcumin and its derivatives are a promising therapy for vector-borne diseases, particularly those transmitted by mosquitoes, like West Nile fever, lumpy skin diseases, malaria, and dengue fever, which are being studied by several researchers who are evaluating its effectiveness in vivo [20,21,22,23].

The most common arboviruses, viruses carried by arthropods that infect people, pets, and livestock, are biologically transferred by mosquitoes. Contagious species of this nature frequently exhibit some anthropophilic traits and are found near hosts that are vulnerable to them. The most prevalent mosquito in both urban and rural regions is *Culex pipiens* (*C. pipiens*) (Diptera: *Culicidae*) [24]. It acts as a vector for numerous viral infections, such as Japanese encephalitis, West Nile fever, and Saint Louis encephalitis, and is the primary vector of filariasis [25]. Mosquitoes are controlled using repellents [26] and chemical pesticides such as organophosphates and pyrethroids [27], which are already being used extensively and have detrimental consequences on non-target animals, such as humans [28,29].

The immune response in mosquitoes includes humoral and cellular aspects; the humoral component responsible for anti-microbial peptide production, while hemolytic cells are responsible for cellular response in addition to its crucial role in both phagocytosis and mosquito encapsulation responses. Hydrogen peroxides (H_2_O_2_) are a natural by-product of aerobic cell metabolism and have broad-spectrum activity against a variety of bacterial endospores, in addition to displaying biodegradablility and nontoxicity toward the environment. [30]. Hydrogen peroxiside has great capability to interact with macromolecules like ribonucleic acid (RNA), deoxyribonucleic acid (DNA) proteins, and lipids, causing drastic structural changes that severely limit insect growth [31]. The majority of cell membranes are crucial for cell growth and replication; lipid peroxidation may be highly harmful and ultimately cause a living system to collapse [32].

Furthermore, excessive amounts of reactive oxygen species (ROS) can easily cause oxidative damage to midgut insect cells, which can further limit nutrient absorption by macerating the enzyme [33]. In biological systems, the reactive oxygen species (ROS) and antioxidants can become unbalanced; this can happen when ROS levels are too high or antioxidant levels are too low, leading to oxidative stress [34,35]. Insects utilize a network of antioxidant enzymes, such as superoxide dismutase (SOD), glutathione reductase (GR), catalase (CAT), Glutathione peroxides (GPx), and in addition to non-enzymatic antioxidants like reduced glutathione (GSH), β-carotene and α-tocopherol, as a self-secretion defense mechanism to guard against oxidative damage [32]. Such secretion of enzymatic, non-enzymatic antioxidant, and macromolecular damage products could be measured as a very good tracer to monitor oxidative stress occurrence [32,36].

Acetylcholinesterase (AChE) is the central nervous system’s key enzyme in insects. It plays a vital part in the hydrolysis of the neurotransmitter acetylcholine, which terminates nerve impulses. The two forms of acetylcholinesterase found in most insects are AChE itself and pseudocholinesterase (BuChE), also known as plasma cholinesterase. AChE inhibition is highly prioritized because of its impact on the neuromuscular system, which results in cholinergic crisis and impacts the entire central nervous system. Instead of laboratory and field trials, bioinformatics and molecular docking is a highly useful method for assisting researchers in selecting the appropriate inhibitor and also in shrinking the probability [37].

Hereon, the current study intends to enhance and comprehend the utilization of curcumin encapsulated in nanostructure lipid carriers and co-delivered with linalool and geraniol mono Terpenes (NLC-Cur-LG) as an insecticide. As essential protective tracers, the oxidative stress alteration was evaluated in the *C. pipiens* larvae in addition to the (i) ROS concentration (H_2_O_2_), (ii) enzymatic antioxidant activity (SOD activity and concentration of ascorbic acid), and (iii) protein carbonyl quantity following NLC-Cur-LG treatment at various doses on *C. pipiens* fourth instars compared to control insects.

## 2. Results

### 2.1. Nanodrug Delivery

#### 2.1.1. Particle Size (DLS) and Zeta Potential and Stability (Z.P)

**Figure 1:**
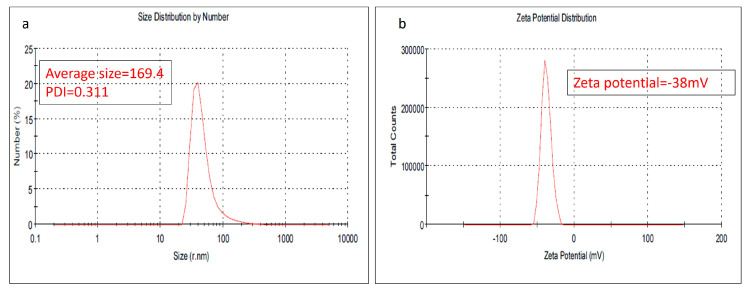
(**a**) The average particle size distribution of the prepared NLC-Cur-LG, (**b**) zeta potential of the prepared NLC-Cur-LG.

As one of the preliminary investigations to check the quality of the nanoparticles, the particle size, measured by dynamic light scattering, showed an average particle size of 169.4 nm with a size variation, measured as the polydispersity index, of 0.311. Meanwhile, the stability of the prepared nanoparticles was evaluated also and the results showed a z.p of −38 mV; Figure 1.

#### 2.1.2. Transmission Electron Microscope (TEM)

**Figure 2:**
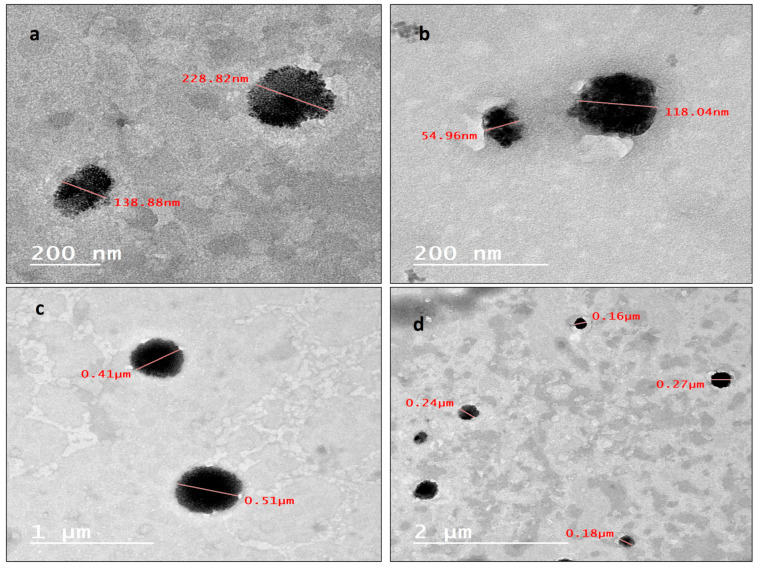
(**a**) Internal morphology by TEM of semi-spherical NLC-Cur-LG nanoparticles; (**b**) internal morphology, investigated using TEM, of spherical NLC-Cur-LG nanoparticles; (**c**) internal morphology, investigated using TEM, of spherical and size-varied NLC-Cur-LG nanoparticles; (**d**) internal morphology, investigated using TEM, of large spherical NLC-Cur-LG nanoparticles.

The internal morphology, investigated using a transmission electron microscope, of the prepared nanoparticles displayed semi-spherical (Figure 2a) and spherical (Figure 2b–d) particles in the range of 200 nm. A noteworthy number of nanoparticles have sizes less than 200 nm, as presented in (Figure 2b,d), such nanoparticles have very small particle sizes ranging from 50 nm to 120 nm, and some other particles have larger sizes, as shown in (Figure 2c), extending to the range of 500 nm.

### 2.2. Insecticidal Evaluation

#### 2.2.1. H_2_O_2_ Concentration

**Figure 3:**
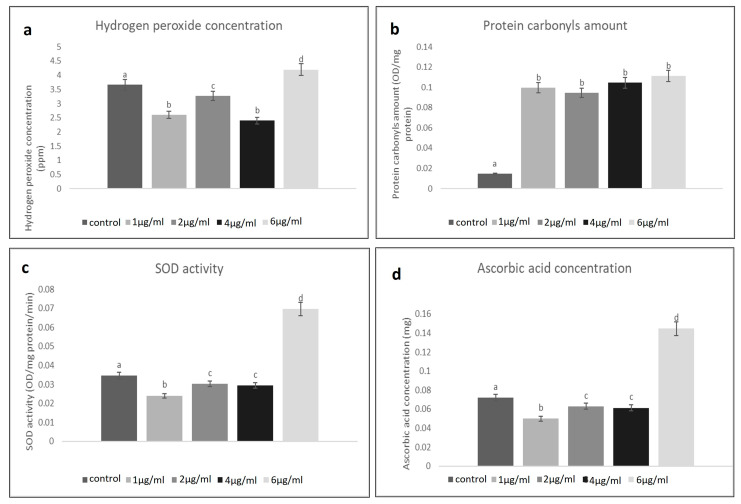
(**a**) Fluctuating hydrogen peroxide levels in the treated mosquito larvae group at different concentrations of NLC-Cur-LG nanoparticles compared to the untreated control; (**b**) elevated levels of protein carbonyls in the treated mosquito larvae group at different concentrations of NLC-Cur-LG nanoparticles compared to the untreated control; (**c**) Fluctuating SOD activities in the treated mosquito larvae group at different concentrations of NLC-Cur-LG nanoparticles compared to the untreated control; (**d**) Fluctuating ascorbic acid level activities in the treated mosquito larvae group at different concentrations of NLC-Cur-LG nanoparticles compared to the untreated control. Bars marked with the same small letters showed no significant difference between different concentration treatments (*p* > 0.05).

The concentration of H_2_O_2_ in the untreated homogenate of the larval tissue of the control groups was found to be 3.5 ppm. At different concentrations of treatment, a fluctuating level of H_2_O_2_ concentration was found. Such changeability assisted as proof of the effects of the NLC-Cur-LG nanoparticles. The concentration of H_2_O_2_ was found to be at its highest at 6 µg/mL (4 ppm), and then it reduced at 2 µg/mL until it almost reached that of the control groups. This continuous decrease was considerable, at 1 µg/mL (2.5 ppm) and 4 µg/mL (2 ppm) less than the control, respectively (Figure 3a). In comparison to the comparable controls, the experimental data revealed significant changes in the activities of the evaluated H_2_O_2_ levels in an attempt to defend and increase immunity.

#### 2.2.2. Protein Carbonyl Amounts

The NLC-Cur-LG nanoparticles treatment significantly increased the protein carbonyl in the *C. pipiens* larvae after 12 h; all the treatments attained a higher level (0.08–0.1 OD/mg) than the control groups (0.01 OD/mg). As seen in Figure 3b, the NLC-Cur-LG treatment increased the total protein levels, which accumulated and may not have been utilized or liberated due to insufficient antioxidant concentrations and the building of a new protective protein due to the effect of H_2_O_2_ disturbance.

#### 2.2.3. Ascorbic Acid Concentration and SOD Activity

After treatment with different concentrations of the NLC-Cur-LG nanoparticles, there were no discernible differences in the SOD activity or concentration of ascorbic acid in the mosquito larvae. A higher level of SOD of 0.06 OD/mg protein was observed, significantly at 6 µg/mL, while at the concentrations 2 and 4 µg/mL, 0.03 OD/mg protein was recorded, which was nearly as the same as the control groups (0.035 OD/mg protein), then the activity decreased to (0.02 OD/mg protein) at 1 µg/mL (Figure 3c,d). This concentration gradient of the produced SOD and ascorbic acid levels affirmed the effective role of the use of NLC-Cur-LG nanoparticles in disturbing their utilization and distribution inside larval body tissues due to raising oxidative stress and lowering in antioxidant concentrations.

#### 2.2.4. Effect of Nanoparticles on the Fourth Instar *C. pipiens*

In response to applying various doses of NLC-Cur-LG nanoparticles (0.5, 0.25, 0.125, and 0.0625 µg/mL), we assessed the effect of the NLC-Cur-LG component on the mortality of the fourth instar *C. pipiens*. The analysis of the dose–mortality response is used to display the data (Table 1). According to the study’s toxicological findings, the NLC-Cur-LG nanoparticles have a fatality rate with an LC_50_ value of 0.649 µg/mL. The experimental larvacidal activity displayed significant physiological changes in terms of the activity of the studied parameters following the administration of the stressor as compared to the comparable controls. The nano-formulated substance has higher virulence against the mosquito larvae than the control, initiating a higher mortality rate, with an LC_50_ of 0.649 mµg/mL, whereas the control exhibited a lower mortality rate, with an LC_50_ of 1.411 mµg/mL. 

#### 2.2.5. Acetylcholinesterase Enzyme Inhibition Assay

An evaluation of the potency of the inhibitory effect of the NLC-Cur-LG towards acetylcholinesterase itself was performed using the enzyme inhibition assay (Table 2). The IC_50_ values of each single component were 14.88, 4.34, 2.42, and 1.95 µg/mL for the curcumin, linalool, geraniol, and NLC-Cur-LG, respectively, compared to the positive control donepezil.

#### 2.2.6. Acetylcholinesterase Activity by Western Blotting

**Figure 4:**
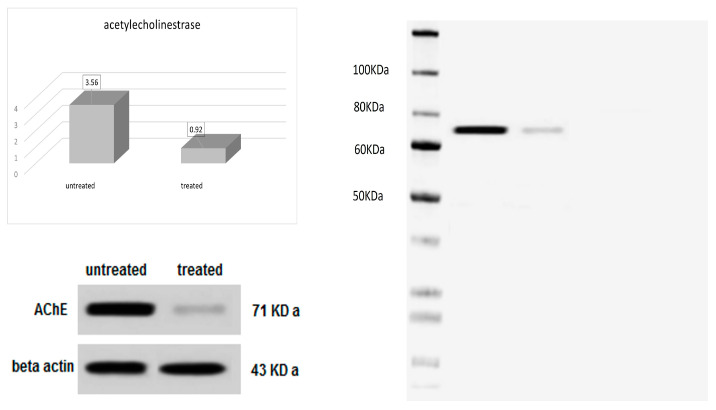
Effect of NLC-Cur-LG nanoformulation on acetylcholinesterase immuno-transmitter enzyme level by Western blotting of treated and untreated groups of *C. pipiens*.

As a confirmation technique, Western blotting was used for the qualitative evaluation of the acetylcholinesterase level of the two groups of *C. pipiens*; the first one was treated with the nanoformulation of NLC-Cur-LG, and the other was an untreated control. The AchE level clearly dropped to a quarter of its original untreated value (Figure 4).

#### 2.2.7. Cytotoxic Effect of NLC-Cur-LG Nanoparticles against Vero and WI38 Normal Cell Lines

**Figure 5:**
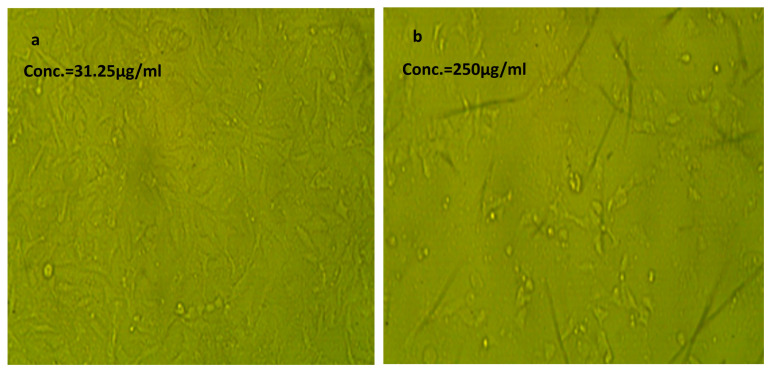
Effect of NLC-Cur-LG against Vero cell line at different concentrations. It examined by inverted microscope at 10×. (**a**) Treatment at concentration of 31.25 µg/mL with very low cytotoxic activity, supported by good number of cells and morphology compared to the untreated control; (**b**) treatment at relatively high concentration of 250 µg/mL; the cell population was affected but still reserved their regular morphology if compared to the untreated control.

**Figure 6:**
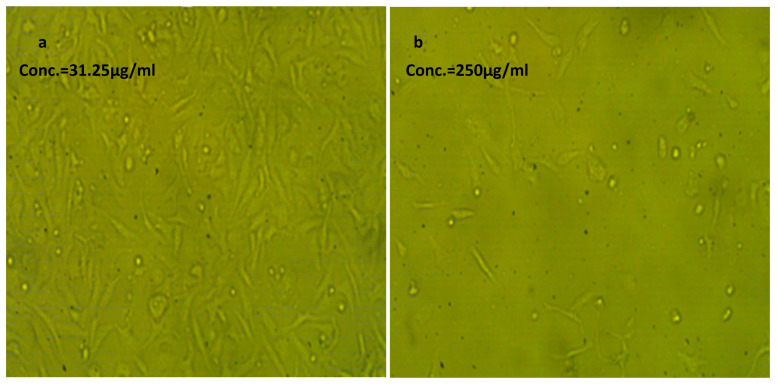
Effect of NLC-Cur-LG against the WI38 cell line at different concentrations. It examined by inverted microscope at 10×. (**a**) Treatment at concentration of 31.25 µg/mL with very low cytotoxic activity, supported by good number of cells and morphology compared to the untreated control; (**b**) treatment at relatively high concentration of 250 µg/mL; the cell population was affected but still reserved their regular morphology if compared to the untreated control.

**Figure 7:**
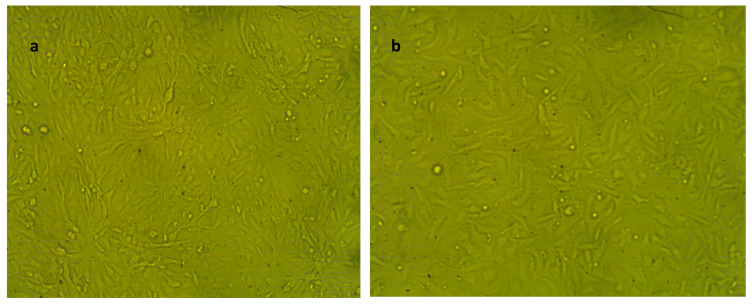
Representative morphology of normal cell lines used in the cytotoxicity test. It examined by inverted microscope at 10×. (**a**) Adherent Vero epithelial kidney normal cell line (untreated control); (**b**) adherent WI38 normal lung fibroblast cell line (untreated control).

The cytotoxicity of the synthesized NLC-Cur-LG against the Vero and wi38 cell lines to show how the synthesized curcumin nps are safe for human use was performed and the results of the optical densities, cell viability, cytotoxicity, and IC_50_s are presented in Table 3 and Table 4. The analysis showed very good results, especially with wi38, with an IC_50_ of 158.15 µg/mL, while the Vero cell line showed an acceptable, moderate IC_50_ of 81.61 µg/mL. The images of the cells shown in Figure 5 and Figure 6 clarifies the relative populations and morphology of the tested cell line treated with the two concentrations of the synthesized nps compared to the untreated cell lines (Figure 7).

### 2.3. Molecular Simulation Docking

**Figure 8:**
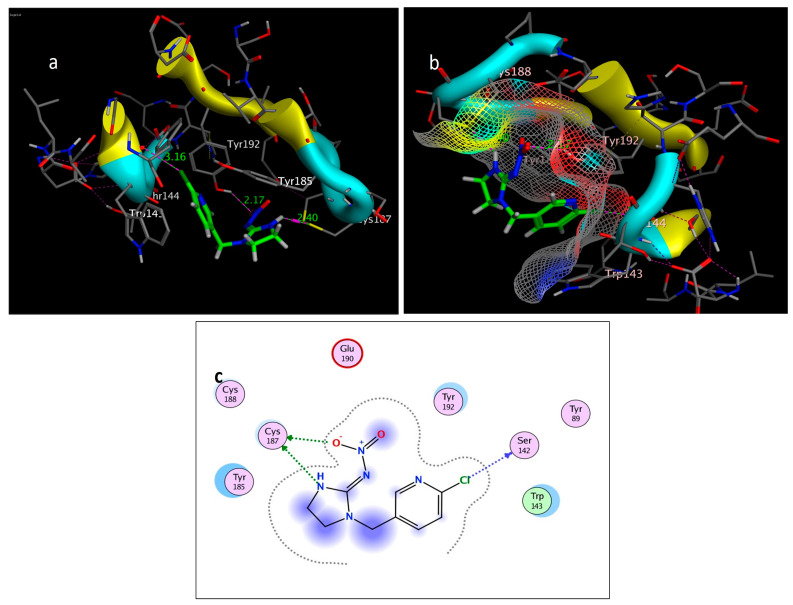
Self-docking of the Imidacloprid (co-crystallized ligand) interior 2ZJU pocket: (**a**) three-dimensional receptor interaction; (**b**) three-dimensional positioning in the receptor pocket; (**c**) two-dimensional interactions.

**Figure 9:**
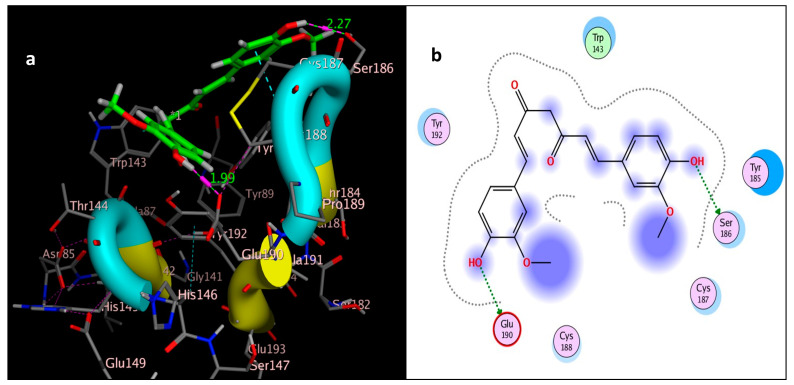
Docking of curcumin (tested ligand) interior 2ZJU pocket: (**a**) three-dimensional receptor interaction of curcumin ligand; (**b**) two-dimensional interactions of curcumin ligand.

**Figure 10:**
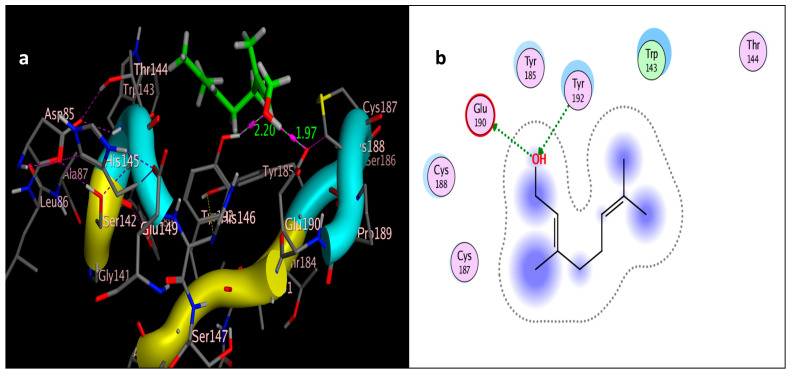
Docking of geraniol (tested ligand) interior 2ZJU pocket: (**a**) three-dimensional receptor interaction of geraniol ligand; (**b**) two-dimensional interactions of geraniol ligand.

**Figure 11:**
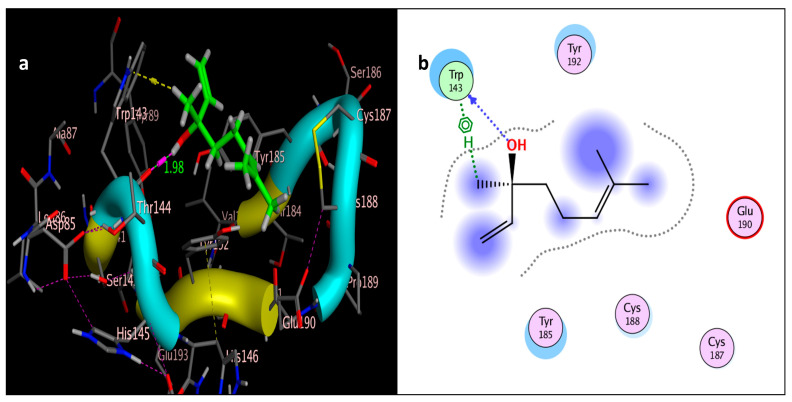
Docking of linalool (tested ligand) interior 2ZJU pocket: (**a**) three-dimensional receptor interaction of linalool ligand; (**b**) two-dimensional interactions of linalool ligand.

**Figure 12:**
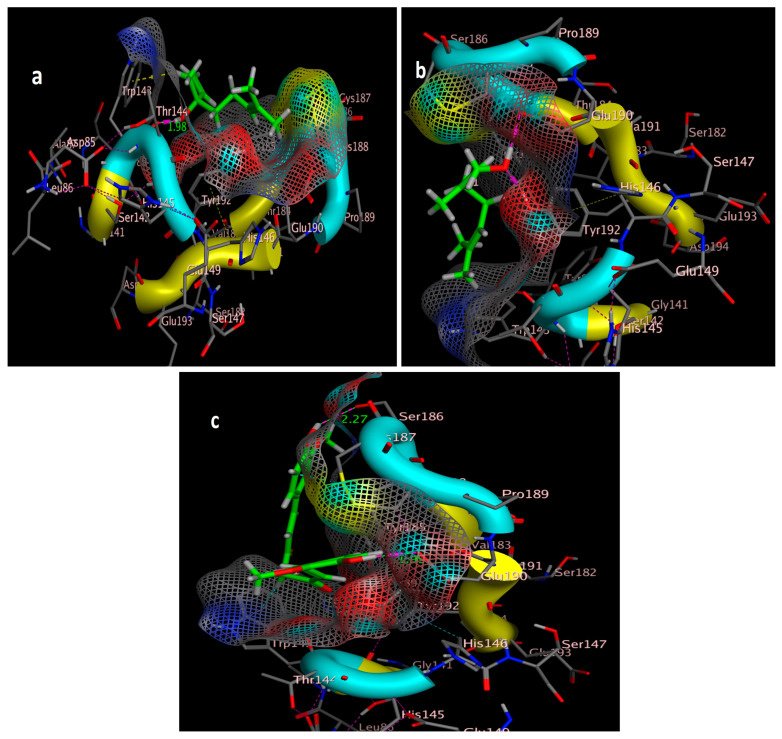
Three-dimensional positioning of the ligand and receptor pocket interior 2ZJU: (**a**) linalool three-dimensional positioning; (**b**) Geraniol three-dimensional positioning; (**c**) curcumin three-dimensional positioning.

The molecular docking study was performed using acetylcholinesterase as the target protein (*Ls*-AChBP (PDB Code: 2ZJU) and curcumin, geraniol, and linalool as the drug ligands, and the results are listed in Table 5. All the simulated compounds were compared to the positive co-crystallized Imidacloprid, which revealed three hydrogen bonds, as shown in Figure 8a,c, while the three-dimensional positioning reference is presented in Figure 8b. Convenient, stable bonds were made by all three tested compounds. Curcumin showed two hydrogen bonds with a bond length of 1.99 and 2.27 Å, as shown in Figure 9a,b. Similarly, geraniol showed two hydrogen bonds with lengths of 1.97 and 2.20 Å as shown in Figure 10a,b. In contrast, linalool interacted weakly, with one hydrogen bond with a bond length of 1.98 Å and pi–pi stacking, as shown in Figure 11a,b. In addition, as a measure of compatibility, the root mean square deviations (RMSDs) were 1.55 Å, 0.823 Å, and 1.51 Å for linalool, geraniol, and curcumin, respectively, and the three-dimensional positioning is presented in Figure 12a–c for linalool, geraniol, and curcumin.

## 3. Discussion

Acetylcholine (ACh) is one of the most crucial excitatory neurotransmitters in the insect central nervous system transmitted from one nerve cell to another. As inhibitory AchE decomposes acetylcholine, by hydrolysis, when released from synaptic vesicles, to its end-products choline and acetate, this means AChE regulates nerve impulse transmission through the cholinergic synapse [38]. Many plant extracts, active ingredients, essential oils, and secondary metabolites like monoterpenoids have been studied intensively for their pest-control, repellent, antifeedant, and ovicidal effects [39,40].

The combination of many monoterpenes like Geraniol and linalool with curcumin in the same formulation may provide a benefit as an AChE inhibitor. The synthesis of NLC-Cur-LG was achieved using the hot homogenization–ultrasonication method. Dynamic light scattering measurements are one of the most important measurements to ensure the nano size of the synthesized nps. The DLS showed an average particle size near 170 nm; such a particle size is very good for the synthesis of a nanostructure lipid carrier [41]. Meanwhile, the polydispersity index, as a measure of the homogeneity of the particle size, was 0.311, confirming that the synthesized NLCs have a wide range of particle size about the mean, and were polydisperse nanoparticles with no sign of monodispersity, as a smaller value for the pdi near the zero point indicates full homogeneity and that all the particles have very similar sizes. The results of the particle size and pdi of the synthesized NLCs within the normal range, as expected, showed that the NLCs have a particle size of 200–400 nm [42], which sometimes extended up to 1 micrometer (1000 nm). The stability profile of all the synthesized nps was controlled in terms of the charge density or zeta potential, being that a more positive or more negative charge is preferable, as this diminishes the attraction forces and enhances the repulsion forces. The NLC-Cur-LG showed a zeta potential of −38 mV, indicating higher stability compared to other nps [43]. Not only the DLS and zeta potential present a convenient measure for the nps’s stability, but also the internal morphology shares the same importance. The synthesized NLC-Cur-LG showed that regular spherical and semi-spherical particles with particle sizes from 50 nm, surpassing 118, 138 and 160 nm, and extending up to 400 nm existed, and this is inconsistent with the pdi results of the polydispersity. Such results are in agreement with those of a synthesized curcumin NLC for breast cancer treatment, which was prepared with a particle size in the range of 100 up to 500 nm, and a pdi from 0.18 up to 0.4 under varying conditions [44].

The inhibitory effect of the synthesized NLC-Cur-LG was studied by two different protocols. The first was the enzyme inhibition assay, the results of which revealed a synergistic effect causing the nanoformulation NLC-Cur-LG to show a very good IC_50_ of 1.95 µg/mL, which was lower than that of the imidacloprid-containing pesticide Skweez, and was very close to the IC_50_ of the inhibitor donepezil (IC_50_ = 1.8 µg/mL). Geraniol and linalool co-delivery in addition to curcumin in one of the nanoformulations generated some type of synergism to inhibit AChE. Specifically, each one of them has high IC_50_s: curcumin 14.88, linalool 4.34, geraniol 2.42, if compared to the nanoformulation NLC-Cur-LG and the positive controls imidacloprid and donepezil. It is noteworthy that geraniol was the only member revealed to have a very high inhibition ability (low IC_50_ =2.42 µg/mL); meanwhile, curcumin displayed a very low inhibition ability; such results match with those found by [45].

As a semi-quantitative method to quantify the existence of distinct enzymes or proteins, Western blotting was used to estimate the change occurring in two different groups of ten insects treated with NLC-Cur-LG and an untreated control. The acetylcholinesterase expression was found to be invasively reduced after treatment with the nanoformulation containing the three active ingredients and the AchE level dropped to a quarter of its original untreated value. The estimation of AChE via an enzyme assay utilizing Western blotting confirmed the inhibitory effect of the synthesized nanoformulation.

The cytotoxic effect of the synthesized NLC-Cur-LG was studied against two types of cell lines: Vero, of the Green monkey kidney cell line; and WI38, of the lung tissue fibroblast cell line, as a preliminary step to ensure the cytotoxic effect and safety. The cytotoxic effect due to the NLC treatment was expressed as the IC_50_. The influence of the NLCs on both cell lines showed high IC_50_ values of 81.6 and 158.1 µg/mL, confirming that the synthesized nanoformulation has a good cytotoxic effect if compared to the pesticide 2,4-phenoxy acetic acid, which has an IC_50_ of 115 ± 4.39 µg/mL after 72 h [46].

For alternative AChE inhibitors rather than cytotoxic organophosphorus and/or carbamate, there is an increasing demand to discover more types of acetylcholinesterase. Molecular docking is one most important artificial intelligence tools that helps to predict the most active candidate from a database. The inhibiting ability of curcumin, linalool, and/or geraniol towards the acetylcholinesterase protein was studied. Imidacloprid (IMI) is one of the neonicotinoid insecticides which work at the nicotinic acetylcholine receptors (nAChARs) in different manners. The nAChARs consist of five subunits: two alpha subunits, beta, gamma, and delta subunits. Hereon, we tried to elucidate the mode of action and or/inhibition capabilities that could be achieved by the active ingredients (curcumin, linalool, and/or geraniol) to replace Imidacloprid. The crystal structure of 2ZJU suggested that the guanidine functional group in IMI preferred to interact with cys187; meanwhile, the nitro group tended to be bonded with the cys187 residue through hydrogen bonding; also, the halogens preferred to overlap and interact with ser142, with a bond length of 2.40, 2.17, and 3.16 Å, respectively. Curcumin and geraniol were furnished with two dipole–dipole hydrogen bonds, where the two hydroxyl groups in curcumin preferred to bind to ser186 and glu190, respectively, while geraniol was bound to the residues try192 and glu190 with a bond length of 1.97 and 2.2 Å. In addition, the terminal methyl in linalool preferred to bind with the residue trp143, but the hydroxyl group hydrogen bonded with the same residue. As an energy-controller parameter, the RMSD values for curcumin and linalool were 1.5104 Å and 1.5504 Å, respectively, and geraniol showed the most stable conformation relative to the positive control IMI and had a very close RMSD value of 0.8234 Å. The RMSD parameter became smaller, indicating that the candidate could replace the co-crystallized ligand (IMI) more easily [47]. The single molecular docking results matches with the results of the enzyme inhibition assay. 

There is a lack of information on the impact of the unique NLC-Cur-LG nanoparticles’ chemistry on oxidative stress parameters, specifically its overall antioxidant capacity and reducing power. This information was used while evaluating the experiment’s results. Insects produce more antioxidant and detoxifying enzymes because of their defense systems against some insecticides [48]. These findings focus on the oxidation of superoxide anion radicals (O^−2^) to oxygen and H_2_O_2_ to explain the function of the SOD enzymes [49]. The highly created H_2_O_2_ is converted to oxygen and water by CAT enzymes [50,51]. When the rate of superoxide anions or H_2_O_2_ breakdown is inadequate, the formation of hydroxyl radicals promotes protein oxidation and denaturation. Many studies have investigated the biomonitoring of pesticide applications using oxidative stress indicators [52,53]. The treatment of NLC-Cur-LG material, which increases the levels of ROS formation, is one internal and external factor that causes the synthesis of H_2_O_2_ in living things to increase. Similar to past findings, the current data has proven a distinguished increase in the H_2_O_2_ generation rate at the highest concentration (NLC-Cur-LG). The treatment of *C. pipiens* larvae causes a rise in ROS production, mainly leading to a decrease in H_2_O_2_ at the lower concentrations than in the control. The results are supported by Fridovich’s [54] theory that when endogenous toxicants enter an insect’s body, they create ROS that raises the levels of H_2_O_2_ [55]. H_2_O_2_ damages cell membranes by oxidizing the proteins that make up those membranes [56]. Proteins may have broken down into free amino acids as a result of the treatment’s stress, which would account for the reported drop in the total protein. The insect may have been trying to manufacture protective proteins to biologically counteract the effects of the H_2_O_2_ if this was the case [57]. Oxidative stress happens when the synthesis and elimination of reactive oxygen species (ROS) are out of equilibrium [58,59]. Protein carbonylation, DNA single-strand breaks, and oxidative damage to lipids are all examples of damage caused by oxidative stress to macromolecules [60,61].

An antioxidant defense system designed to reduce both enzymatic and non-enzymatic oxidative damage can reduce cellular macromolecular activities that cause oxidative damage. Several insect species are subject to oxidative damage due to a number of biotic and abiotic situations, as well as severe environmental stressors such as fungal, bacterial, and viral illnesses, insecticides, low or high temperatures, and others. The SOD enzymes become active when oxygen is present. Ascorbate, the major non-enzymatic antioxidant that, together with ascorbate peroxidase, aids aphids in eliminating damaging H_2_O_2_, substantially decreases as oxidative stress increases, according to Łukasik et al. [62]. These enzymes support superoxide being changed into oxygen and hydrogen peroxide. The levels of reactive oxygen species are regulated by SOD enzymes (ROS). The ascorbic acid and SOD activity in mosquito larvae reduced in conjunction with the varied dosages. Furthermore, the larvae of *Spodoptera exigua* subjected to high humidity displayed considerably reduced levels of SOD, peroxidase, and CAT activity compared to a control [63]. These data emphasize the multiple ways that increased insect mortality contributes to the depletion of the antioxidant system. It also supports the use of additional oxidative stress markers or the mosquito larvae’s antioxidant response as a signal for NLC-Cur-LG nanoparticles’ material. It was also discovered to be a novel technique for perhaps using NLC-Cur-LG to upset the tissues of mosquito larvae.

## 4. Materials and Methods

### 4.1. Synthesis of Nanostructure Lipid Carrier Encapsulated Curcumin (NLC-Cur-LG)

Curcumin (Cur) 97%, oleic acid 90%, stearic acid 97%, polysorbate 20 (tween 20), sodium taurocholate 96%, sodium glycocholate 97.5%, hydrogen peroxide 29%, and deionized water were procured from Alfa Aesar (Thermo Fisher Scientific, Dreieich, Germany), whereas linalool and geraniol were procured from Acros Organics (Thermo Fisher Scientific, Dreieich, Germany). Skweez (contains Imidacloprid 34%) insecticide was purchased locally from Semadak (Damanhur, El Beheira governorate Egypt). All chemical reagents were utilized without being purified, with the exception of Skweez insecticide, which was lyophilized to produce a powder.

### 4.2. Biochemical Assays

Biochemical enzymes, levels of SOD and GST, MDA content, and hydrogen peroxide levels were evaluated using colorimetric chemical kits supplied by Sigma-Aldrich Chemie GmbH (Sigma Aldrich, Merck, Darmstadt, Germany), Eschenstrasse 5, D-82024 TAUFKIRCHEN, and protocols and the chemical reagent quantities were mixed as recommended by Sigma-Aldrich (Sigma Aldrich, Merck, Darmstadt, Germany).

### 4.3. Synthesis of Encapsulated Nanostructure Lipid Carrier

The synthesis of lipid carrier nanoparticles encapsulating Cur, co-delivering linalool and geraniol monoterpenes (NLC-Cur-LG) was performed using a homogenization procedure in accordance with Radwan et al. [48] with crucial modifications. The synthesis protocol included three main steps: briefly, the aqueous solution was prepared in the first beaker (B1) as follows: 6 mL deionized water, 0.1 mL butanol as co-surfactant, and 5 mL Tween 20 were well combined with a thoroughly agitated pre-warmed mixture of equivalent quantities of sodium glycocholate and sodium taurocholate (each one 0.35 g) dissolved in 10 mL water and maintained at 45 °C.

In beaker number two (B2), the solid and liquid lipids are mixed in a ratio of 1:2, respectively, as follows: 0.7 gm of stearic acid, 0.7 gm oleic acid, and 0.010 gm (100 mg) of curcumin were placed on hotplate stirrer, and 0.5–0.7 mL of solvent mixture was added. In order to prepare a stock solvent mixture, chloroform and methanol were mixed at a ratio of 3:1 (*v*/*v*), the temperature was slowly increased to 85 °C, at which point all of the curcumin was dissolved and all of the stearic acid had molted, and the heating process was extended for another 7 min, or till the solvent had completely evaporated. 

The temperature of (B2) was allowed to decrease to 70 °C (by means of an infrared thermometer to show the temperature changing and warming was continued to this degree); the molten substance was a distinct yellow color.

In the third beaker (B3), exactly 0.7 gm of oleic acid and 0.02 gm (200 mg) of both linalool and geraniol (each one 100 mg) were injected underneath the oleic acid using a micropipette. Stirring at 150 rpm for 2 min reduced the volatility.

The contents of the beaker (B3) were transferred to the beaker (B2), and mechanical stirring was performed at 250 rpm for one minute at the same temperature, 70 °C (overheating was avoided). The beaker (B1) was swiftly mixed with the mixture and stirred for two minutes at 600 rpm. The resulting emulsion was retained in a 50 mL falcon tube at 10 °C after being sonicated for 15 min using probe sonication (VCX 750 & 13 mm probe). For estimating the cytotoxic effect of the synthesized nanoparticles against normal cell lines and the acetylcholinesterase enzyme inhibition assay, a very small amount of the synthesized nps was freeze-dried using a lyophilization process under vacuum conditions and −50 °C to obtain the nps transformed from the dispersed phase to the solid phase.

### 4.4. Characterization of Nanostructure Lipid Carrier

#### 4.4.1. The Particle Size (DLS), Polydispersity (PDI), and Zeta Potential

The dynamic light scattering technique (DLS) was used to measure the radius and polydispersity index (PDI). The room temperature of 25 °C was chosen as the measurement setting, and the angle was set at 173°. The zeta potential (z.p) was explored by determining the frequency change of the dispersed light caused by laser beam irradiation at a scattering angle of 12°. The average size, zeta potential, and PDI measurements were performed by (Zeta sizer Nano ZS, Malvern Instruments Ltd. Cambridge, UK). At the zero day preparation, 10 mg of the nano dispersion solution was dispersed in 20 mL double distilled water for 5 min at room temperature (25 °C) using probe sonication.

#### 4.4.2. Transmission Electron Microscope (TEM)

The visualization of the internal structural shape and the produced nanostructure lipid carrier were performed using field transmission microscopy (HR-TEM, JSM-7100F). The pictures were captured using a JEOL JEM2100-115 high-resolution transmission electron microscopy equipment, with the electron-accelerating voltage ranging from 100 to 200 kV. The NLC sample was produced as follows: 1 µL of NLC was substantially dissolved in deionized water(1:200 *v*/*v*) and dropped on a 200-mesh carbon-coated grid; after 5 min, a cellulose filter was used to extract the excess liquid NLCs. A drop or two of phosphotungstic acid (PTA) was applied to the grid for 10 s to facilitate negative staining, and extra PTA was then discarded by filter paper absorption.

### 4.5. In Vitro Cytotoxicity Effect of NLC-Cur-LG

The cytotoxicity assessment of the produced nanoformulation was performed using the human lung fibroblast cell lines WI38, American type culture collection, CCl-75 (Sigma Aldrich, Merck, Germany). The cell line WI38 was grown in RPMI media with 10% fetal bovine serum (FBS) and 100 units/mL antibiotics (streptomycin and penicillin, each at 100 g/mL to inhibit the growth of bacteria). The incubation procedure was carried out in a CO_2_ incubator with a 37 °C temperature and a 5% humidity rate. Further, the cell lines were transferred into 96-well plates, to be seeded at a density of 1.0 × 10^5^ per well and incubated for 48 h. The cells were subsequently treated with several doses of the NLC-MB-MT nanoformulations (1000, 500, 250, 125, 62.5, and 31.25 µg/mL) and after reaching the suitable conditions for the desired cell confluency, the cells were re-incubated for 48 h at the same humidity and temperature settings. Each seeded well received 20 μL of MTT solution at 5 mg/mL conc., and the wells were incubated for an additional 4 h to evaluate the cell viability and, subsequently, the IC_50_. The mitochondrial succinate dehydrogenase enzyme in viable cells, or live cells, converts the yellow soluble tetrazolium salt into insoluble purple crystals of formazan compound, at which time, the non-viable cells die. The sharpness of the purple formazan was assessed using colorimetry after 100 L of dimethyl sulfoxide (DMSO) was added to each well to ensure that all of the formazan was thoroughly dissolved. The colorimetric test was measured at ƴ_max_ = 570 nm using a plate reader (EXL 800, California, CA, USA). (Treated samples/Untreated sample) × 100 was used to calculate the relative cell viability (%). The IC_50_ was computed mathematically based on the conversion of cell viability numbers into toxicity.

### 4.6. Acetylcholinesterase Inhibition Assay

To evaluate AChE enzyme inhibition, a colorimetric test kit (Biovision, Cairo, Egypt) was utilized. The curcumin nanoformulation was sonicated for five minutes in order to produce a serial dilution, and then it was diluted with distilled water. Precisely, a 96-well plate was filled with 10 µL of nanoformulation sample and the positive control, donepezil, which had been liquified in a suitable solvent such as DMSO. The following procedure yields the AChE enzyme solution: After dilution of reconstituted AChE 25 times (i.e., mix 2 L of AChE with 48 L of AChE assay buffer), add 10 µL of dissolved AChE to every well having tested chemicals and negative and positive (solvent) controls. Each well volume should be adjusted to 160 μL using the AChE assay buffer. Protect from light and incubate for 10–15 min at room temperature. We used a microplate ELISA reader (Bioline, Thane, Maharashtra, India) with a wavelength set to 450 nm to measure the absorbance of 10 samples.

### 4.7. Western Blotting

Following tissue homogenization in all groups, the ReadyPrepTM total protein extraction kit (Bio-Rad Inc., California, CA, USA) was applied to each sample according to manufacturing guidelines. For quantitative protein analysis, the Bradford Protein Assay Kit (Bio-Rad Inc., CA, USA) was utilized. To determine the protein content in the sample, a Bradford assay was performed following the instructions of the manufacturer. Each protein sample was loaded with an equal quantity of 2*Laemmli sample buffer, which included 4% SDS, 10% 2-mercaptoethyl, 0.004% bromophenol blue, 20% glycerol, and 0.125 M Tris HCl.

The pH was examined and corrected to a somewhat acidic level of 6.8. The prior solutions were heated for 5 min at 95 °C to confirm full protein denaturation before loading on polyacrylamide gel electrophoresis. The TGX Stain-FreeTM FastCastTM Acrylamide Kit (SDS-PAGE) (Bio-Rad Inc., CA, USA) was used to prepare polyacrylamide gels. The SDS-PAGE TGX Stain-Free FastCast was performed following the manufacturer’s specifications. The gel was constructed in a transfer sandwich from bottom to top (filter paper, PVDF membrane, gel, and filter paper). The sandwich was put in a transfer tank containing 1x transfer buffer (25 mM Tris, 190 mM glycine, and 20% methanol). Using the BioRad Trans-Blot Turbo, in order to allow protein bands to move from gel to membrane, the blot was then run for seven min at 25 V. The membrane was blocked for 1 h at room temperature in tris-buffered saline with 3% bovine serum albumin (BSA) and Tween 20 (TBST) buffer. As directed by the manufacturer, primary antibodies were dissolved in TBST. Every primary antibody solution was left overnight to incubate against the blotted target protein at 4 °C. The blot was washed with TBST three to five times for 5 min. The blotted target protein was treated in the HRP-conjugated secondary antibody solution for one hour at room temperature (Goat anti-rabbit IgG-HRP-1 mg Goat mab—Novus Biologicals). The chemiluminescent substrate Clarity TM Western ECL substrate (Bio-Rad Inc., CA, USA) was used for the blot as described by the manufacturer. The chemiluminescent signals were collected using a CCD camera-based imager. The band intensity of the target proteins on the ChemiDoc MP imager was measured using image analysis software by protein normalization against the control sample beta actin (housekeeping protein). 

### 4.8. Molecular Docking of Acetylcholineesterase Enzyme

#### 4.8.1. Source of the Objective Protein

The binding affinity of the examined drugs against the Ls-AChBP binding site was investigated using a theoretical technique called molecular docking to evaluate if the chemicals interact with the protein’s binding site. The docking study used the well-known three-dimensional crystal structure of the Lymnaea stagnalis acetylcholine binding protein Ls-AChBP (PDB Code: 2ZJU) with no homology modelling because the acetylcholine esterase nAChR does not have a three-dimensional crystal structure in the Protein Data Bank (https://www.rcsb.org/structure/2ZJU). Water molecules, as well as any heteroatoms, were removed from the protein retrieved in PDB format, leaving just chain A. 

#### 4.8.2. Energy Minimization

Curcumin, linalool, and geraniol were drawn in CAMBRIDGESOFT CHEMOFFICE 2015 Professional 15.0.0 programme and saved in Mol format. The most stable conformation was generated by performing the energy minimization step using the default data set of Amber12; gradient convergence of 0.01 kcal/mol was achieved by using the EHT forcefield. The energy minimization and interaction simulation was conducted using the Molecular Operating Environment (MOE_2015.10), which was set up on a 64-bit Intel (R) Core (TM) i5-2400 CPU @ 2.40 GHz, 8 GB RAM system.

#### 4.8.3. Docking Procedure

Following the upload of Ls-AChBP (PDB Code: 2ZJU), for both ligand and protein preparation, the co-crystallized drug (imidacloprid) or reference drug was color-coded in green to make it easy to differentiate. To retrieve the co-crystallized ligand binding sites directly, the surfaces and maps option was used to recognize the binding sites. In order to allow for flexible ligand–rigid receptor docking, the ligands and proteins were fully prepared, and the docking was completed using the default settings via the “Rotate Bonds” option. Furthermore, the scoring function was altered to the London G with triangle matcher replacement, 40 conformers were adjusted instead of the best score ligand’s automated choice conformer, and low energetically stable configuration were preserved. The top five conformers score of ligand–receptor docking were then shown by two- and three-dimensional ligand–receptor interactions [64]. The docking results are summarized into one table taking into consideration the most stable interaction pose, scoring energy (kcal/mol), RMSD(Å), and the bond length (Å). Three- and two-dimensional docking interactions were captured, and green was used to allow the ligand under docking investigation to be easily distinguished; intermolecular hydrogen bonding and π-π staking (aromatic) were labeled in magenta and yellow; meanwhile, the loops, helixes, etc. of protein structures were colored automatically by the software and some rendering set ups were made for best presentation of the protein. 

### 4.9. Insecticidal Evaluation

#### 4.9.1. Laboratory Rearing of *C. pipiens*

*C. pipiens* was obtained from Giza Governorate in 1985 and colonized at Cairo University’s Entomology Department’s laboratory after being morphologically recognized according to taxonomic keys of Harbach and Knight [65]. A enclosed white enamel pan containing fresh food (protein 48.0%, ash 11.0%, oil 8.0%, 2.0% fiber, and moisture 6.0%) was filled with hundreds of newly born larvae. Dead larva were removed every day in order to ensure the greatest raising conditions possible. Evaporation-lost water was replenished with fresh, distilled water. Using a collection sieve, pupae were removed from breeding cages and placed in plastic pots (7 cm × 6 cm) half-filled with deionized water before being transferred to adult breeding cages. *C. pipiens* mosquitoes were reared in mesh-screened wooden cages with holes for insertion of pupae and other daily routine tasks such as feeding, egg removal, and cage cleaning. Both males and females were given a tiny Petri dish containing a cotton pad soaked with a 10% sucrose solution as a carbohydrate source. To avoid fungal infection, the sucrose solution was changed every day. At night, blood feeding was provided by attaching a domestic pigeon to the cage’s top, which was provided three to four days after the female emerged, and sugar was withheld for 12 h before blood feeding was started. The gravid females were placed in 250 mL plastic cups that were partially full with water to facilitate ovipositing while increasing the relative humidity in the cage at the same time. Every day, oviposited egg rafts were gathered and delicately moved to white enamel pans which were partially filled with water using a tiny paintbrush. To stop other species from ovipositing, these pans were covered until the eggs hatched.

#### 4.9.2. Insecticidal Effect and Enzyme Assessment

The *C. pipiens* larvae were provided by Cairo University’s Entomology Department, Faculty of Science. The larvae were housed in a plastic cage 20 × 20 × 10 cm^3^ for every 200 larvae and new food was supplied every day. Concentrations of nanoformulation desired for NLC-Cur-LG were made by dissolving the stock solution in various beakers at varied concentrations (1, 2, 4, 6 µµg/mL) in double distilled water, then larvae were added to each beaker. Untreated insects (control) were submerged in fresh distilled water for 24 h and their mortality was recorded. The larvae were then encased in mesh and homogenized in a 5 mL phosphate buffer mixture containing 50 mM phosphate buffer, 5 mL of 0.05 mM CaCl_2_, 10 mL of 0.1% Triton 100, and a total amount of 100 mL of deionized water. Following homogenization, the samples were centrifuged at 2000 g for 15 min at 4 °C. According to Levine et al. [66] a pool of ten insects was utilized to test bioassays with three separate replicates and the amount of protein carbonyls. The protein standard was bovine serum albumin (BSA) fraction V (Sigma-Aldrich, St. Louis, MO, USA), and the Bradford technique was used in spectrophotometry to determine the total protein content of the samples [67]. Meanwhile, SOD activity was measured according to Misra and Fridovich’s technique [68]. The reaction mixture is as follows: 87 mL of supernatant from the relevant tissue, 35 mL of EDTA (10 mM), 402 mL of a sodium carbonate buffer (200 mM; pH 10.0), and 2835 mL of freshly generated epinephrine (15 mM). A UV/Vis Jenway-7305 spectrophotometer (Bibby Scientific Limited, Staffordshire, UK) was used to assess the absorbance of each biomarker, and the protein concentration was expressed as OD/g protein/min to indicate activity of SOD. Ten grams of the sample were homogenized in a blender to provide a sample extract for the ascorbic acid content assay. After that, the substance was added to a 250 mL conical flask along with 50 mL of an acetic acid solution containing 5% metaphosphoric acid. The final 50 mL of the phosphoric acid solution was added back into the flask. The filtrate was collected and tested for vitamin C after filtering the solution using Whatman filter paper. A small amount of bromine solution was mixed into the filtered sample solution. To eliminate the bromine solution, a few drops of thiourea solution were added to the sample solution. After that, 1 mL of a 2,4 DNPH solution was added to the sample solution. The coupling process was started with a 2,4 DNPH solution. To allow the reaction to complete, the sample solution and standards were maintained at 37 °C for 3 h. After three hours of chilling in an ice bath, 5 mL of H_2_SO_4_ was added. Thus, the colored solutions were ready to have their absorbance measured at 560 nm. The spectrophotometric approach of Junglee et al. [69] was used to estimate the H_2_O_2_ concentration. Simply defined, the extraction–colorimetric process consisted of a homogenization step with PBS, pH = 7.0, mixed with 0.5 mL KI (1 M), 0.25 mL Trichloroacetic acid (TCA) (0.1% (*w*:*v*)), and 1 mL samples centrifuged at 12,000× *g* at 4 °C for 15 min to detect absorbance wavelength at 240 nm.

## 5. Conclusions

As well as using artificial intelligence simulations, in addition, nanotechnology helps to accurately predict the activity of and merge more than one active ingredient into a nanoformulation. Linalool and geraniol monoterpenes were merged with curcumin in one NLC nanoformulation as an insecticidal control. As a reflex reaction, paradoxical oxidative stress enzyme levels in addition to other biochemical variations in the mosquito larval tissues were as a result of the NLC-Cur-LG application. Acetylcholinesterase enzymes were differently assessed using two protocols and together reinforced the enzyme level diminishment due to the application of the nanoformulation. The cytotoxic evaluation against the Vero and WI38 normal cell lines were evaluated, and initial cytotoxicity outcomes were obtained that may help additional investigations of the synthesized nanoformulation, NLC-Cur-LG, in the field of application.

## Figures and Tables

**Table 1:** LC_50_ of NLC-Cur-LG nanoparticles against fourth instar of *C. pipiens* at different concentrations.

Treatment	n ^a^	Slope ± SE	LC_50_ (95% CI)	χ^2 b^	Df	*p*
NLC-Cur-LG	750	0567 ± 0.127	0.649 (0.369–3.27)	4.095	10	<0.001
control	750	0.399 ± 0.157	1.411(1.051–1.812)	3.635	10	<0.001

^a^ fifty larvae per replicate, three replicates per concentration, four concentrations, and a control per bioassay. ^b^ Pearson’s χ^2^ goodness-of-fit test on the probit model (α = 0.05).

**Table 2:** AChE enzyme inhibitory effect IC_50_ of the synthesized NLC-Cur-LG nanoformulation and its components.

Compound	Curcumin	Linalool	Geraniol	Skweez Pesticide (Imidocloprid 34%)	NLC-Cur-LG	Donepezil
IC_50_ (µg/mL)	14.88	4.34	2.42	2.11	1.95	1.8

**Table 3:** Cytotoxicity effect of NLC-Cur-LG against normal Vero cell line.

	Con.	O. D			Aver O. D	St. Dev.	S. E	Viability	Toxicity	IC_50_ (µg/mL)
Vero		0.694	0.681	0.689	0.688	0.0065574	0.003786	100	0	81.61 ± 3.2
NLC-Cur-LG	1000	0.051	0.059	0.062	0.057333	0.0056862	0.003283	8.333333	91.66667	
	500	0.042	0.055	0.056	0.051	0.0078102	0.004509	7.412791	92.58721	
	250	0.065	0.039	0.052	0.052	0.013	0.007506	7.55814	92.44186	
	125	0.14	0.119	0.156	0.13833333	0.0185562	0.010713	20.10659	79.89341	
	62.5	0.425	0.395	0.378	0.39933333	0.0237978	0.01374	58.04264	41.95736	
	31.25	0.5	0.535	0.523	0.51933333	0.0177858	0.010269	75.4845	24.5155	

**Table 4:** Cytotoxicity effect of NLC-Cur-LG against normal wi38 cell line.

	Con.	(Optical Density) O. D			Aver O. D	St. Dev.	S. E	Viability	Toxicity	IC_50_ (µg/mL)
Wi38		0.566	0.559	0.583	0.56933333	0.0123423	0.007126	100	0	158.15 ± 5.2
NLC-Cur-LG	1000	0.029	0.032	0.018	0.02633333	0.0073711	0.004256	4.625293	95.37471	
	500	0.033	0.042	0.065	0.04666667	0.0165025	0.009528	8.196721	91.80328	
	250	0.123	0.114	0.141	0.126	0.0137477	0.007937	22.13115	77.86885	
	125	0.28	0.41	0.43	0.37333333	0.0814453	0.047022	65.57377	34.42623	
	62.5	0.392	0.59	0.48	0.48733333	0.0992035	0.057275	85.59719	14.40281	
	31.25	0.489	0.555	0.59	0.54466667	0.0512868	0.02961	95.66745	4.332553	

**Table 5:** Docking study of acetylcholinesterase (Ls-AChBP (PDB Code: 2ZJU)). control is Imidacloprid.

Compound	Interactions	Residue	Type	Distance (Å)	Score (kcal/mol)	RMSD (Å)
imidacloprid	3	Cl → Ser142 (Backbone)	H-bonding	3.16	−4.6308	0.8343
		NO_2_ → Cys187	H-bonding	2.17		
		NH → Cys187	H-bonding	2.40		
curcumin	2	OH → Ser186	H-bonding	2.27	−5.5592	1.5104
		OH → Glu190	H-bonding	1.99		
Geraniol	2	Try192 → OH	H-bonding	1.97	−4.0244	0.8234
		OH → Glu190	H-bonding	2.20		
linalool	2	OH → Trp143 (Backbone)	H-bonding	1.98	−4.1315	1.5514
		CH_3_ →Trp143 (benzene ring)	π-π interactions			

## Data Availability

Data are contained within the article.
